# Facile preparation of a novel biogenic silver-loaded Nanofilm with intrinsic anti-bacterial and oxidant scavenging activities for wound healing

**DOI:** 10.1038/s41598-020-63032-5

**Published:** 2020-04-09

**Authors:** Hassan Bardania, Reza Mahmoudi, Hamed Bagheri, Zeinab Salehpour, Mohamad Hassan Fouani, Bita Darabian, Seyed Sajjad Khoramrooz, Ali Mousavizadeh, Majid Kowsari, Seyyed Ebrahim Moosavifard, Gunna Christiansen, Danesh Javeshghani, Mohsen Alipour, Mohammad Akrami

**Affiliations:** 1 Cellular and Molecular Research Center, Yasuj University of Medical Sciences, Yasuj, Iran; 2 Medicinal Plant Research Center, Yasuj University of Medical Sciences, Yasuj, Iran; 3 Clinical Research Development Unit, Imamsajad Hospital, Yasuj University of Medical Sciences, Yasuj, Iran; 4 Faculty of Interdisciplinary Science and Technology, Tarbiat Modares University, Tehran, Iran; 5 Department of Nanobiotechnology, Faculty of Biological Sciences, Tarbiat Modares University, Tehran, Iran; 6 Social Determinants of Health Research Center, Yasuj University of Medical Sciences, Yasuj, Iran; 7 Department of Advanced Medical Sciences & Technologies, School of Medicine, Jahrom University of Medical Sciences, Jahrom, Iran; 8 Department of Biomedicine, Aarhus University, 8000 Aarhus C, Denmark; 9 Department of Pharmaceutical Biomaterials and Medical Biomaterials Research Center, Faculty of Pharmacy, Tehran University of Medical Sciences, Tehran, Iran

**Keywords:** Biomedical materials, Nanoparticles

## Abstract

To eliminate the microbial infection from an injury site, various modalities have been developed such as dressings and human skin substitutes. However, the high amount of reactive oxygen species, microbial infection, and damaging extracellular matrix remain as the main challenges for the wound healing process. In this study, for the first time, green synthesized silver nanoparticles (AgNPs) using *Teucrium polium* extract were embedded in poly lactic acid/poly ethylene glycol (PLA/PEG) film to provide absorbable wound dressing, with antioxidant and antibacterial features. The physicochemical analysis demonstrated, production of AgNPs with size approximately 32.2 nm and confirmed the presence of phytoconstituents on their surface. The antibacterial assessments exhibited a concentration-dependent sensitivity of *Staphylococcus aureus* and *Pseudomonas aeruginosa* toward biosynthesized AgNPs, which showed a suitable safety profile in human macrophage cells. Furthermore, oxidant scavenging assays demonstrated exploitation of plant extract as a reducing agent, endows antioxidant activity to biogenic AgNPs. The formation of PLA/PEG nanofilm and entrapment of AgNPs into their matrix were clearly confirmed by scanning electron microscopy. More importantly, antibacterial examination demonstrated that the introduction of biogenic AgNPs into PLA/PEG nanofibers led to complete growth inhibition of *P. aeruginosa* and *S. aureus*. In summary, the simultaneous antioxidant activity and antimicrobial activity of the novel biogenic AgNPs/PLA/PEG nanofilm showed its potential for application as wound dressing.

## Introduction

Skin, the body’s largest organ, interfaces between the external environment and the body, protecting it against pathogens and dehydration. Upon wound injury of the skin, the wounded site provides a suitable milieu for bacterial growth owing to its moist, warmth, and nutritious environment. Therefore, in order to facilitate wound tissue regeneration, it is crucial to provide a better tool to eliminate microbial infections from the injury site^1,2^. To meet this purpose, various modalities have been devised such as dressings, antimicrobials, growth factors, and human skin substitutes^3^.

Nanofibers prepared from renewable polymers, due to their extremely high specific surface area, porosity and excellent pore interconnectivity, excellent performances in cell adhesion, migration, proliferation, differentiation, and the analogous physical properties of the extracellular matrix (ECM) have attracted considerable attention over the past two decades^4^. Such characteristics allow structured nanofibers to promote hemostasis without the use of a hemostatic agent, maintain an adequately humid environment for the wound by facilitating oxygen permeation and allowing fluid accumulation, protect the wound effectively against bacterial penetration, and be easily functionalized with therapeutic compounds^5^. Wound healing and dermal reconstitution capacity of various biopolymers such as fibrinogen, chitosan, collagen, poly (caprolactone), gelatin, poly (urethane), poly (lactic acid) (PLA) have been extensively evaluated^6,7^. PLA is one of the choices for use in wound dressings for several reasons such as biocompatibility, biodegradability, non-toxicity, absorbability and reasonable price^8^. However, PLA’s lack of flexibility makes it impossible to implement PLA solely in wound dressings^9^. On the other hand, poly (ethylene glycol) (PEG) is a flexible, nontoxic, nonimmunogenic polymer, which renders it suitable to be implemented to overcome PLA’s shortcomings^10^.

Antimicrobial agents play a vital role in reducing bacterial loads. However, it is highly expedient for an antimicrobial agent to have an extended efficacy, thereby preventing bacterial re-colonization and proliferation. Among the most studied methods to extend the antimicrobial activity, is the integration of antimicrobial agents into sustained-release delivery systems^11^.

There is a good deal of evidence that biocidal metals such as silver and copper slowly release their cations in trace amounts that are toxic to bacteria, a characteristic which has been implemented in thwarting wound’s contamination. Since ancient times, in its ionic or nanocrystalline form, silver has been exploited as an antimicrobial agent against a broad spectrum of pathogenic micro-organisms; a detailed history of silver usage has been well documented in literature^12^.

Nanotechnology, in recent years, has rendered it possible to fabricate various forms of AgNPs^13^. The unique small size of AgNPs is characterized with a larger surface area-to-mass ratio, which results in better contact with bacteria and hence achieves a higher antimicrobial efficacy^14^. However, in practice the efficacy of AgNPs, especially smaller ones, is hampered by their ease of aggregation; surface passivator reagents are needed to prevent aggregation^15^. Unfortunately, organic passivators such as thiophenol, thiourea and mercaptoacetate are environmentally hazardous. One of the approaches to avoid AgNPs aggregation, hence retain their antimicrobial efficacy, is to incorporate them into biodegradable polymers^16,17^. Several reports exist on the incorporation of AgNPs into mats or dressings in various forms: powders, foams, hydrogels, polymeric films and meshes; each form claims to have certain advantages, but bactericidal efficacy of silver is common among them all^18–21^.

It is noteworthy to mention that traditional chemical reductants implemented in AgNPs synthesis, such as hydrazine, dimethyl hydrazine, NaBH_4_, etc., are environmentally hazardous and will bring potential environmental risks. However, numerous studies have reported the utilization of medicinal plants to synthesize and stabilize metallic nanoparticles, very particularly AgNPs, via the green synthesis method; a method considered to be cost-effective, environment-friendly and easily scaled up for large-scale synthesis of nanoparticles^22–24^. Furthermore, this method does not require high pressure, temperature, and energy. Therefore, alternatives less hazardous and economically viable methods are being explored. Many reports are available on the biogenesis of AgNPs using several plant extracts^25^.

Compounds that prevent the oxidation of biological molecules through constraining the propagation of the oxidizing chain reactions are considered “antioxidants”^26^. It has been demonstrated that “antioxidants” facilitate wound-healing. Reactive oxygen species (ROS) production is one of the defense mechanisms common to inflammation cells against invading pathogens. Wound site is characterized by high ROS levels, which might result in severe tissue damage and even lead to neoplastic transformation decreasing the healing process rate by damages in cellular membranes, DNA, proteins and lipids. Those adverse effects of ROS could be inhibited through introducing “antioxidants” to the wounded site. Antioxidants ‘sacrifice’ themselves through scavenging free radicals and becoming “less reactive” and hence less harmful than the radicals themselves; through this mechanism, antioxidants enhance healing wounds process^27–30^. Hence, the introduction of biogenically synthesized AgNPs onto PLA/PEG composite film can endow the material with good free radical scavenging capacity.

Byun *et al*. incorporated α-tocopherol as a natural antioxidant into PLA/PEG film. They observed about 6.5 times the enhancement of the antioxidant activity of the film^31^. Iglesias Montes *et al*. reported that the incorporation of Umbelliferone and lignin nanoparticles into PLA/PEG bilayer film increases its antioxidant properties^32^. On the other hand, a number of studies have designed PLA/PEG film contained antimicrobial agents for various applications. Barbosa *et al*. demonstrated that the addition of terpinen‐4‐ol to PLA/PEG reduces the viable Aggregatibacter actinomycetemcomita cells of biofilm^33^. Another study indicated that the incorporation of magnesium oxide nanoparticles into PLA/PEG enhances the antibacterial efficacy of the film^34^. Turalija *et al*. used plasma technology to silver nanoparticles deposition on PLA/PEG films for enhancing its antibacterial properties^35^. Vasile *et al*. also prepared PLA/PEG film containing rosemary extract as an antioxidant agent and chitosan as an antimicrobial agent for subcutaneous implantation^36^.

Here, for the first time, we have incorporated of biogenic AgNPs into PEG/PLA film, to simultaneously endow antibacterial and antioxidant properties with low cytotoxicity to nanofilm for overcoming wound healing process’s challenges. There is no report for the incorporation of biogenic AgNPs in PLA/PEG film for wound dressing application. In the present study, we report the synthesis of AgNPs, using *Teucrium polium*’s (*T. polium*) hydro-alcoholic extract. *T. polium* due to its unprecedenteds antibacterial, antioxidant, antiviral and antifungal properties have been used in traditional medicine (TM) for various types of pathological conditions, such as inflammations and gastrointestinal disorders^37^. The hydro-alcoholic extract of *T. polium* was exploited to reduce silver ions, in order to impart the antibacterial and antioxidant properties of *T. polium* to AgNPs. Subsequently, biogenically produced AgNPs with augmented antibacterial properties were incorporated into PLA/PEG porous composite films to form antimicrobial porous Ag nanofilm as a wound dressing. Morphology and antibacterial activities of porous Ag nanofilm were investigated. We anticipate that the Ag nanofilm wound dressing with free radical scavenging capacity would enhance the wound healing process. Moreover, this study proposed a low-cost and facile method for preparation of Ag nanofilm as a potential wound dressing.

## Materials and Methods

### Materials

Methanol (CH_3_OH, 99.9%), silver nitrate (AgNO_3_), nutrient agar, ferric chloride hexahydrate (FeCl_3_, 6H_2_O), iron (II) sulfate tetrahydrate (FeSO_4_.4H_2_O), dichloromethane, polyethylene glycol 300, ascorbic acid and Mueller-Hinton agar (MHA) were purchased from Merck (Germany). 2,2-diphenyl-1-picrylhydrazyl (DPPH), 3-(4, 5-dimethylthiazol-2-yl)-2, 5-diphenyltetrazolium bromide (MTT) and 2,4,6-Tripyridyl-s-Triazine (TPTZ) were purchased from Sigma (Germany) and poly lactic acid (PLA) from Zhejiang Hisun Biomaterials (China). The RAW264 macrophage cell line was purchased from National Cell Bank, Pasteur Institute of Iran, Iran and Dulbecco’s Modified Eagle’s Medium (DMEM), Fetal Bovine Serum (FBS), 1% Penicillin-Streptomycin from Gibco (Paisley, UK). All aqueous solutions were prepared using double distilled water. All reagents used were of analytical grade. Clinical strains of *Pseudomonas aeruginosa*^38^ and *Staphylococcus aureus*^39^ bacteria were isolated from hospitalized patients by our group, which have been characterized as antibiotic-resistant strains.

### Extract preparation

*T.polium* is a wild-growing flowering plant, found abundantly in South-Western Asia, Europe and North Africa. Fresh whole *T. polium* plants were collected from Kohgiluyeh and Boyer-Ahmad province, Iran. The total hydroalcoholic extract was obtained by percolation method using 80% ethanol^40^. Extracts were concentrated using a vacuum rotary evaporator (Heidolph, Germany) and then freeze-dried (Christ Alpha 2–4, Osterode, Germany) to obtain dry powder.

### Synthesis of AgNPs

*T. polium* extract was used as a reducing agent for AgNPs synthesis. Briefly, 1 mL of the crude extract (100 mg/ml) was added to 40 mL of AgNO_3_ solution (3 × 10^−3^ M) and mixed at 80 °C temperature for 35 minutes. The reaction mixture was then centrifuged at (15000 rpm for 20 minutes) to remove unreacted components.

### Assessing SPR properties of AgNPs

The optical properties were analyzed using UV-Vis spectroscopy by monitoring the electron spectra of the samples employing a CE 7250 UV-Vis spectrophotometer (Cecil Instrument, UK). The spectral bandwidth ranged from 300 to 700 nm at a 1 nm wavelength resolution.

### FT-IR spectroscopy and X-ray diffraction analysis

The presence of functional groups on the surface of AgNPs was assessed using Fourier transform infrared (FT-IR) spectroscopy. FT-IR spectra were recorded from KBr pellets using Thermo NICOLET IR 100 Spectrometer (Thermo Electro Corporation, USA).

X-ray diffraction (XRD) analysis was conducted by PW-1730 system (Philips, Netherlands) using monochromatic Co Kα radiation (λ = 1.7889 Å) operated at 30 mA and 40 kV at 2θ angle pattern; scanning was done in the region of 20°–80°. The diffracted intensities were recorded from 30 °C to 80 °C.

### Particle size and zeta potential analysis

Particle size and Zeta Potential of AgNPs were evaluated by dynamic light scattering (DLS) instrument (Zetasizer Nano ZS, Malvern Instruments Ltd. Worcestershire, England).

Size distribution and shape of particles were examined using transmission electron microscopy (TEM) (JEM-1010; JEOL, Tokyo, Japan) and scanning electron microscopy (SEM) (KYKY-EM3200, KYKY Technology Development Ltd, China).

### Analysis of nanofilm physicochemical properties

Thermogravimetric analysis (TGA) and differential thermal analysis (DTA) spectra have been recorded in a temperature ranging from room temperature to 600 °C using a simultaneous thermal system (STA504, New Castle, DE USA). Measurements were recorded under nitrogen flow with a heating rate of 10 °C min^−1^.

The mechanical properties of the nanofilms were evaluated by a universal tensile test equipment (Instron Co., USA). Samples (with various PLA/PEG ratios) with rectangular shape and thickness of about 20 μm were used for analysis under ambient condition.

### Antioxidant assay of nanoparticle

#### Free radical scavenging activity on DPPH

Antioxidant and radical scavenging potential of the as-formed AgNPs were assessed by the ability of the nanoparticles to scavenge the stable purple colored free radical 1, 1-diphenyl-2-picrylhydrazyl (DPPH) and convert it into yellow colored Diphenyl picryl hydrazine. For that, 1 ml of the sample was mixed with 1 ml of DPPH solution (0.1 mM) and kept in the dark for 15 minutes. The degree of decolorization was assessed spectrophotometrically at 517 nm employing a CE 7250 UV-Vis spectrophotometer (Cecil Instrument, UK). Ascorbic acid and methanol were used as positive (standard) and negative controls, respectively. All experiments were repeated three times and the percentage of scavenging activity was calculated according to Eq. (1).

1 $${\rm{Scavenging}}\,{\rm{activity}}\,( \% )=[({\rm{Abs}}\,{\rm{control}}-{\rm{Abs}}\,{\rm{sample}})/{\rm{Abs}}\,{\rm{control}}]\times 100$$

Where Abs control and Abs sample represent the absorbance of DPPH radical with methanol and tested samples/standard, respectively.

### FRAP assay

Ferric ion Reducing Antioxidant Power assay (FRAP) was also used to measure the total antioxidant power of freshly prepared AgNPs. This method is based on the ability of the sample to reduce Fe^3+^ to Fe^2+^ ions and the subsequent reduction ferric-tripyridyltriazine (Fe^3+^-TPTZ) complex to the ferrous (Fe^2+^ -TPTZ) form accompanied with the formation of an intense blue color having an absorption maximum at 593. Briefly, 0.5 ml of AgNP was mixed with 1.5 ml of FRAP reagent and the absorbance measured at 593 nm after 5 min incubation at room temperature employing a CE 7250 UV-Vis spectrophotometer (Cecil Instrument, UK). FRAP reagent was prepared by mixing 25 ml of 300 mM acetate buffer (pH 3.6), 2.5 ml of 10 mM TPTZ solution and 2.5 ml of 20 mM FeCl_3_ solution in a 10:1:1 ratio. The standard curve was prepared using FeSO_4_.4H_2_O at a concentration ranging between 100 and 500 µg and used to determine the antioxidant potential of the sample.

### Anti-bacterial assay of AgNPs

Agar Disc-diffusion and MTT assays were applied to assess the antibacterial activity of AgNPs against gram-positive species (*Staphylococcus aureus*) and gram-negative species (*Pseudomonas aeruginosa*) bacteria.

Bacterial strains were grown overnight in Müller-Hinton (MH) broth medium, were streaked on Müller-Hinton (MH) agar plates. Bacteria were left to form a confluent lawn of cells over the surface of the plate. Paper discs (6 mm in diameter) sterilized by autoclaving were dipped in AgNPs solution (1000 µg/ml). AgNPs laden discs were air-dried under sterile conditions, and placed onto the seeded top layer of the MH agar plates. Then, the plates were incubated at 37 °C for 24 h and subsequently examined for evidence of zones of inhibition, which appear as a clear area around the wells.

The viability of bacterial cells was tested by measuring the reduction of the yellow colored methylthiazolyldiphenyl-tetrazolium bromide (MTT) to its purple-colored insoluble formazan. For this purpose, 100 µL bacterial suspension (*Staphylococcus aureus* and *Pseudomonas aeruginosa*) grown overnight in MH broth medium in sterile 96-multiwell cell culture plates were treated with different concentrations of AgNPs (31.25, 62.5, 125, 250, 500 and 1000 µg/ml); free MH broth medium and AgNPs synthesized via tri-sodium citrate were used as negative and positive controls, respectively. Afterward, the MTT solution was added to each bacterial suspension (0.5 mg/mL per well) and incubated for 4 hours in the dark. DMSO solution was used to dissolve formazan crystals formed by viable bacterial cells. Absorbance was read at 570 nm using a microtiter plate reader (BioTek ELx800; BioTek Instruments Inc., Winooski, VT, USA). The percentage of viable cells was deduced according to followed equation (Eq. 2).

2 $${\rm{Cell}}\,{\rm{viability}}\,( \% )=({\rm{Abs}}\,{\rm{of}}\,{\rm{sample}}/{\rm{Abs}}\,{\rm{of}}\,{\rm{control}})\times 100$$

### Analysis of safety and biocompatibility of AgNPs

Colorimetric MTT assay was conducted to assess the *in vitro* cytotoxicity of AgNPs on macrophage-like cell line, RAW264. In a 96-well plate, seeded RAW264 cells (4 × 10^5^ cells/well) were grown in Dulbecco’s Modified Eagle Medium (DMEM) supplemented with 10% FBS, penicillin (100 units/mL) streptomycin sulfate (100 µg/mL) in a humidified atmosphere of 5% CO_2_ for 24 hours. Subsequently, cells were treated for another 24 hours with different concentrations of AgNPs and plant extracts (31.25, 62.5, 125, 250 and 500 µg/ml). Then, MTT (0.5 mg/mL) in phosphate-buffered saline (PBS) was added to each well and further incubated at 37 °C, 5% CO_2_ for 3 hours. Finally, media was removed, and DMSO was added to solubilize formazan crystals before recording the absorbance at 570 nm using a microtiter plate reader (BioTek ELx800; BioTek Instruments Inc., Winooski, VT, USA). The percentage of cell viability was calculated by the following equation (Eq. 3).

3 $${\rm{Cell}}\,{\rm{viability}}\,( \% )=({\rm{Abs}}\,{\rm{of}}\,{\rm{sample}}/{\rm{Abs}}\,{\rm{of}}\,{\rm{control}})\times 100$$

### Preparation of AgNP loaded PLA/PEG nanofilms

Solvent volatilization method was implemented in order to prepare flexible polylactic acid)/polyethylene glycol (PLA/PEG) (1:1) film^41^. Briefly, equal amounts of PLA and PEG (500 mg) were dissolved in dichloromethane (DCM) (40 ml) and stirred at 40 °C for 30 minutes with a magnetic stirrer. At the end, a completely homogeneous, transparent and bubble-free solution was obtained. Eventually, the solution was cast onto a glass petri dish (8 cm in diameter) and dried at ambient temperature for 24 hours to form an unloaded film. The solution was then placed in a vacuum oven at 50 °C for 24 hours to dry completely. In order to prepare AgNP loaded PLA/PEG films (AgNP nanofilm), a procedure similar to the unloaded film preparation was followed with a difference in that AgNPs (50 and 100 mg) will be added to the solution. Then the PLA/PEG/AgNPs solutions were stirred for 60 minutes by a magnetic stirrer. Similarly, the solutions were cast onto a glass petri dish and dried at ambient temperature for 24 hours to form, 5% and 10% AgNPs films.

### Morphology of AgNPs containing nanofilms

The morphology of synthesized AgNPs containing PLA/PEG nanofilm was analyzed using scanning electron microscope (Model KYKY-EM3200).

### The bactericidal capacity of AgNP nanofilms

Liquid culture test was exploited to evaluate the antimicrobial activity of the Ag nanofilm. The bactericidal capacity of Ag nanofilm was tested against *S. aureus* and *P. aeruginosa* bacterial strains. Firstly, about 100 µL of bacterial suspensions (OD ~ 0.2) were seeded in a 96-well plate. Then, square samples of sterilized Ag nanofilm (1, 2, 3, 4, 5, 6 mm^2^) containing different percentages of AgNPs (0, 5 and 10%) were added to each well. Plates were then incubated at 37 °C with continuous shaking (150 rpm). Finally, after 24 h of incubation, absorbance at 610 nm was measured using a microtiter plate reader (BioTek ELx800; BioTek Instruments Inc., Winooski, VT, USA) and the percentage of bacterial growth inhibition was calculated by the following equation.

$${\rm{Bacteria}}\,{\rm{inhibition}}\,( \% )=({\rm{Ic}}-{\rm{Is}})/{\rm{Ic}}\times 100$$

Where Ic and Is are the absorbance average of control and sample groups, respectively.

### Statistical analysis

All experiments were performed three times, and the outcomes were presented as mean ± standard deviation (SD). Analysis of variance (ANOVA) with the Tukey post-hoc analysis was used to determine the statistical significance for antibacterial activity and cytotoxicity evaluation. P < 0.05 was considered as statistically significant. GraphPad Prism 5 was applied for the statistical analysis.

## Results and Discussion

### Design of antibacterial and anti-oxidant nano composite

**Figure 1:**
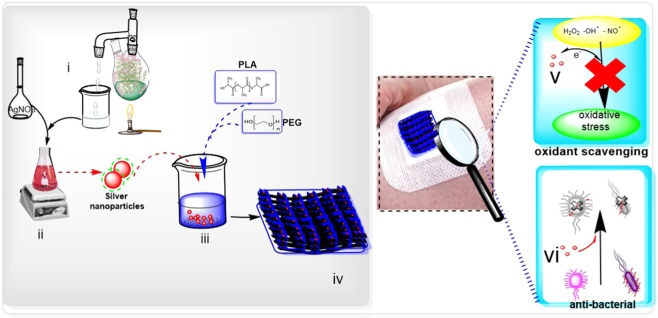
Facile preparation of silver-loaded nanofilm for wound healing (**i**) preparation of plant extract; (**ii**) Fabrication of AgNPs using plant extract as a reductant for silver nitrate; (**iii**) Blending of polyethylene glycol (PEG) and Polylactic acid (PLA) with AgNPs; (**iv**) Formation of a film of PLA/PEG/Ag upon heating at 40 °C; (**v**) PLA/PEG/Ag films were able to scavenge the free radicals species of oxygen which observed in wound sites (**vi**) PLA/PEG/Ag films were able to kill the gram-positive and gram-negative bacteria.

In this study, we have exploited *T.pollium* as a green reductant for bio-fabrication AgNPs. This synthesis approaches not only endows antioxidant feature to the AgNPs but also augments their antibacterial activity. In the next step, the designed bi-functional particle constitutes the main reinforcement for PLA/PEG/Ag nano composite to improve the wound healing features of PLA/PEG co-polymer. The designed super-porous Nano-film to transport oxygen and provide a good stiffness and elasticity (Fig. 1).

### Biofabrication and optimization of AgNPs

**Figure 2:**
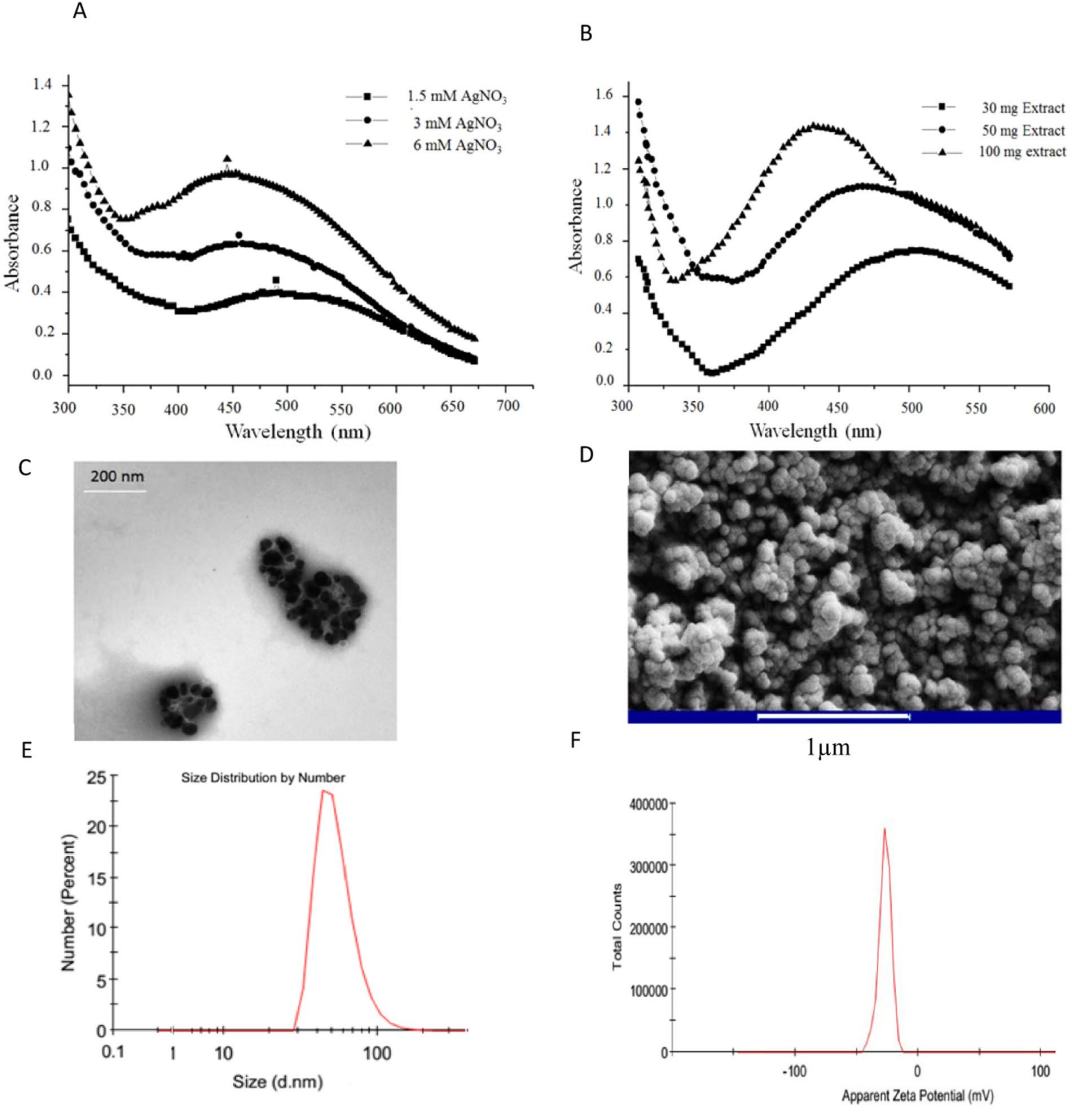
Bio-fabrication and physicochemical characterization of AgNPs. (**A**) UV/visible spectra of AgNP synthesized with various concentrations of AgNO_3_ (**B**) and various amounts of *T. polium* extract (**C**) Transmission electron microscopy (**D**) Scanning electron microscopy micrographs of green synthesized AgNPs using *T. polium* extract (**F**) Size and (**E**) zeta potential of AgNPs measured using DLS analysis.

Due to the intense surface plasmon resonances (SPRs) of colloidal metal nanoparticles^42^, it is possible to confirm the formation of AgNPs using UV-visible spectroscopy. Features such as size, shape, etc. are among the factors that affect the position of SPRs^43^. As shown in Fig. 2a,b, the UV-Vis spectra revealed a broad SPR band in the range of 350–600 nm, indicating formation of colloidal AgNPs. Moreover, the SPR position and intensity demonstrated that the formation of AgNPs is affected by the concentration of silver nitrate and plant extract where the efficiency of AgNPs formation improved upon increasing concentration of silver nitrate and plant extract.

The observed concomitant increment in the relative intensity of the SPR peaks along with increasing silver salt concentration or *T. polium* extract amount, implies an increment in the amount of Ag° nanoparticles as a result of the increased reduction of Ag^+^ (Fig. 2a). The observed SPR in *T. polium* extract: AgNO_3_ samples reveals the reduction of silver ions (Ag^+^) to the metallic AgNPs (Ag°) in the present system. Moreover, the fact that SPRs maximum peaks did not exceed 500 nm, indicates that most of the AgNPs obtained are smaller than 100 nm^44^. Moreover, the UV-Vis analysis of AgNPs demonstrated that the increase of Ag ions and plant extract concentrations during the synthesis process, led to a shift in the AgNPs spectral peak to lower wavelengths. This result could presumably be attributed to the increase of nanoparticles’ nucleation seeds and in turn production of nanoparticle with smaller size. Moreover, based on previous reports AgNPs with small size show their SPR peaks at lower wavelengths compared to AgNPs with large size^45^. Furthermore, addition of fresh *T. polium* extract to silver nitrate solution during stirring at room temperature gradually changed the yellow color of extract intro red, indicating the Ag ion reduction and AgNPs formation.

The analysis of shape and size of AgNPs nanoparticles using TEM and SEM methods revealed that nanoparticles were spherical in shape with approximate size of 32.2 ± 6 nm (Fig. 2c,d).

Size and zeta potential of AgNPs were determined using DLS. Particle size distribution curve depicts that AgNPs obtained are poly-dispersed in nature (PDI 0.154) with average diameter ∼54.64 nm (Fig. 2e) and the corresponding average zeta potential value is −27 ± 5.03 mV (Fig. 1f). DLS technique measures hydrodynamic size of the nanoparticles, which corresponds to the real diameter plus the diameter of electrostatic potential around nanoparticles, therefore it is larger than obtained data from TEM and SEM analysis^46,47^. Moreover, the intense negative charge of AgNPs suggested that fabricated nanoparticles are very stable^48^.

### AgNPs Surface Content Analysis

**Figure 3:**
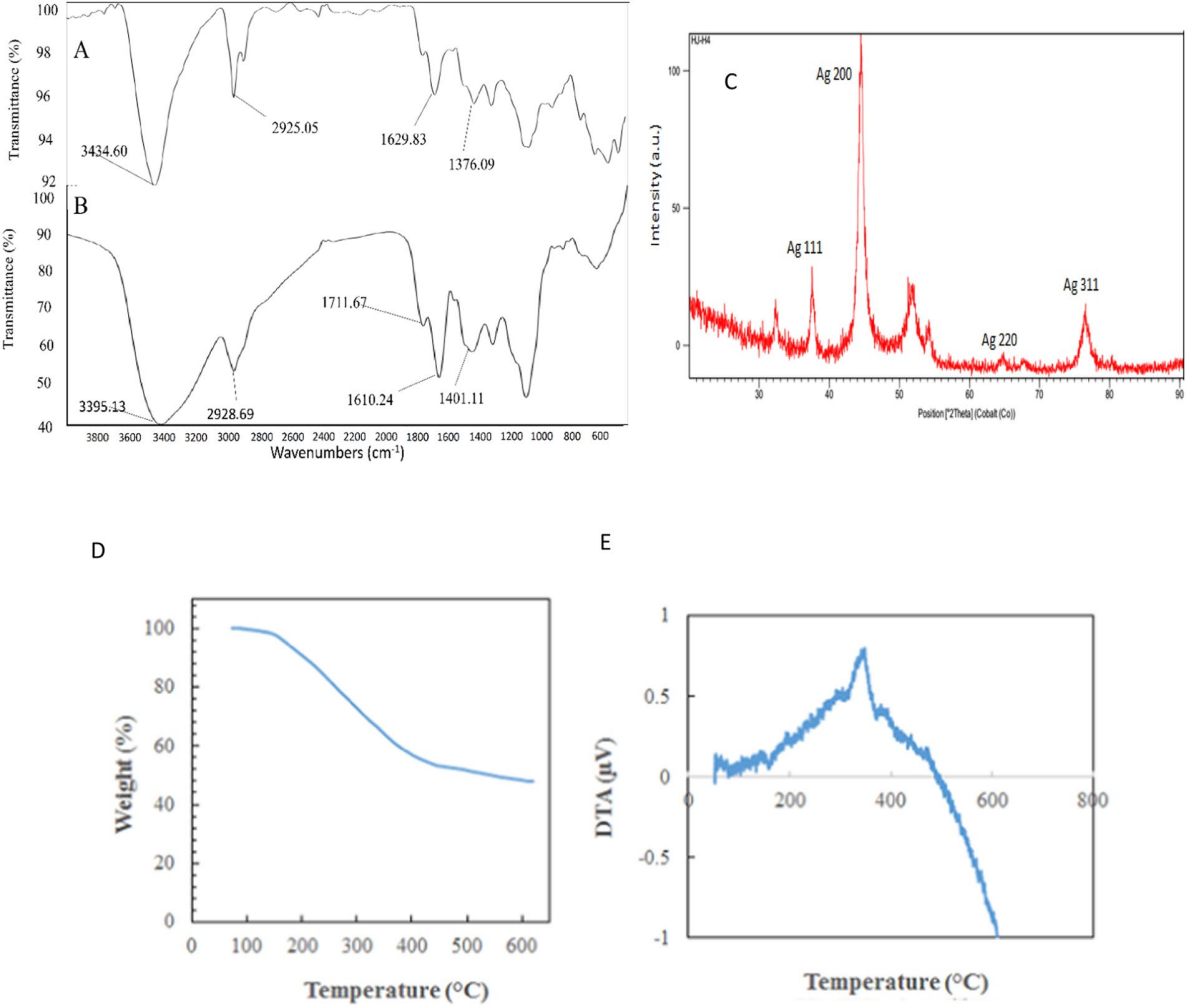
Analysis of surface and content of AgNPs. (**A**) FTIR spectrum of vacuum-dried powder of AgNPs (**B**) FTIR spectrum of vacuum-dried powder of *T. polium* extract (**C**) XRD patterns of AgNPs synthesized using an extract of *T. polium*. (**D**) Thermogravimetric analysis of green synthesized AgNPs. (**E**) Differential thermal analysis curves of green synthesized AgNPs.

FT-IR measurements were carried out to identify the presence of functional groups in biomolecules responsible for capping/stabilization of AgNPs. The observed intense bands were compared with standard values to identify the functional groups (Fig. 3a,b). The comparison of FT-IR spectra of plant extract and AgNPs indicate a shift in peaks 3395.13–3434.60 (bonds corresponding to NH or OH stretching), 2928.69–2925.05 (bond corresponding to CH stretching, alkanes), 1610.24–1629.83 (characteristic of amino acids containing NH2 groups or amide I band) and 1401.11–1376.09 (corresponding to CH deformation, ketones, and esters). These results suggested that amides, hydroxyl, carboxyl, amino groups, and amino acid residues are involved in the bio-reduction of Ag+ ions and are responsible for capping/stabilization AgNPs. *T. polium* extract is composed of various biologically active phytoconstituents including α-thujone, geraniol, caryophyllene, phytosterols, flavones, terpenes, fenchone, cineole, α-Thujone, borneol, which supports the FTIR findings^37^. Furthermore, the crystalline nature of the biosynthesized AgNPs was demonstrated by X-ray crystallography. The XRD pattern of the synthesized AgNPs is shown in Fig. 3c. Four Bragg’s reflections planes in the 2θ correspond to the planes of (1 1 1), (2 0 0), (2 2 0) and (3 1 1) which can be indexed according to the facets of face-centered cubic crystal structure of silver^49^.

The solid-state characterization of AgNPs was also performed using thermogravimetric and differential thermal analyses (TG/DTA). The mentioned characterizations include determination of loss on drying, thermal stability, phase transition temperatures, and water is bound or unbound. TG/DTA measures the change in mass of a sample as a function of temperature (TG) with the temperature difference between sample and inert reference material as a function of temperature (DTA). As shown in Fig. 3d, the obtained thermogravimetric analysis curve verified the thermal stability of green synthesized AgNPs in an inert atmosphere. The TGA curves also displayed the three significant weight loss steps. The first step was around 150 °C and displays a slight weight loss of ∼2%; it has to do with the drying. The second step extends from 150° to 450 °C and displays a major steady-state weight loss ∼45%; it could be attributed to the loss of volatiles and desorption of bioorganic phytocompound. The third weight loss (∼1.5%) step at about 600 °C is due to more thermostable phytochemicals such as oils.

Another thermal analysis, DTA reports changes in temperature of the sample with respect to reference when subjected to heat. DTA thermogram, revealing a peak at 350 °C, infers that the weight loss due to decomposition of organic residues is an exothermic reaction (Fig. 2e and S9). Both TGA and DTA results suggest that AgNPs were stable up to 150 °C.

### Antioxidant activity

The introduction of antioxidant compounds into wound dressing has been demonstrated to present a positive effect on the wound healing process through the regulation of ROS overproduction^27^. The free radical scavenging activity of *T. polium* extract, green synthesized AgNPs and chemically synthesized AgNP was evaluated by FRAP and DPPH radical scavenging assays. As shown in Fig. 3a,b, according to FRAP and DPPH assays, free radical scavenging activity of green synthesized AgNPs increased in a concentration-dependent manner, while chemically synthesized AgNP did not show a high significant antioxidant property. In addition, the antioxidant potential of AgNPs was similar to *T. polium* extract. These results confirmed the presence of various bioactive compounds of the extract on the surface of AgNPs. Based on the fact that silver can exist in two states of oxidation (Ag^+^ and Ag^2+^), depending on the reaction environment, AgNPs may be able to quench free radicals by donating or accepting electrons^50^.

It is demonstrated that the potent DPPH radical scavenging potential of AgNPs might be ascribed to their ability to donate electrons or hydrogen ions to neutralize the unstable DPPH free radicals in the reaction medium. However, the results also indicated that the marginal increase in antioxidant activity of green synthesized AgNPs, compared to the plant extract suggested that the plant extract itself is responsible for the majority of the antioxidant activity and AgNP is not contributing much to the antioxidant activity. On the other hand, it is well documented that flavonoids and phenolic compounds of *T. polium* contribute directly to antioxidative action^37^. Flavonoids and phenolic contents are characterized with redox properties, which allow them to act as reducing agents, hydrogen donors, and singlet oxygen quenchers, hence, exhibit antioxidant activities^26^. Bahramikia S *et al*. reviewed the total flavonoid and phenolic of *T. polium*^37^. Thus, one of the reasons behind the introduction of biogenically synthesized AgNPs into the PLA/PEG dressing was their good antioxidant activity.

### Antibacterial activity assessment

**Figure 4:**
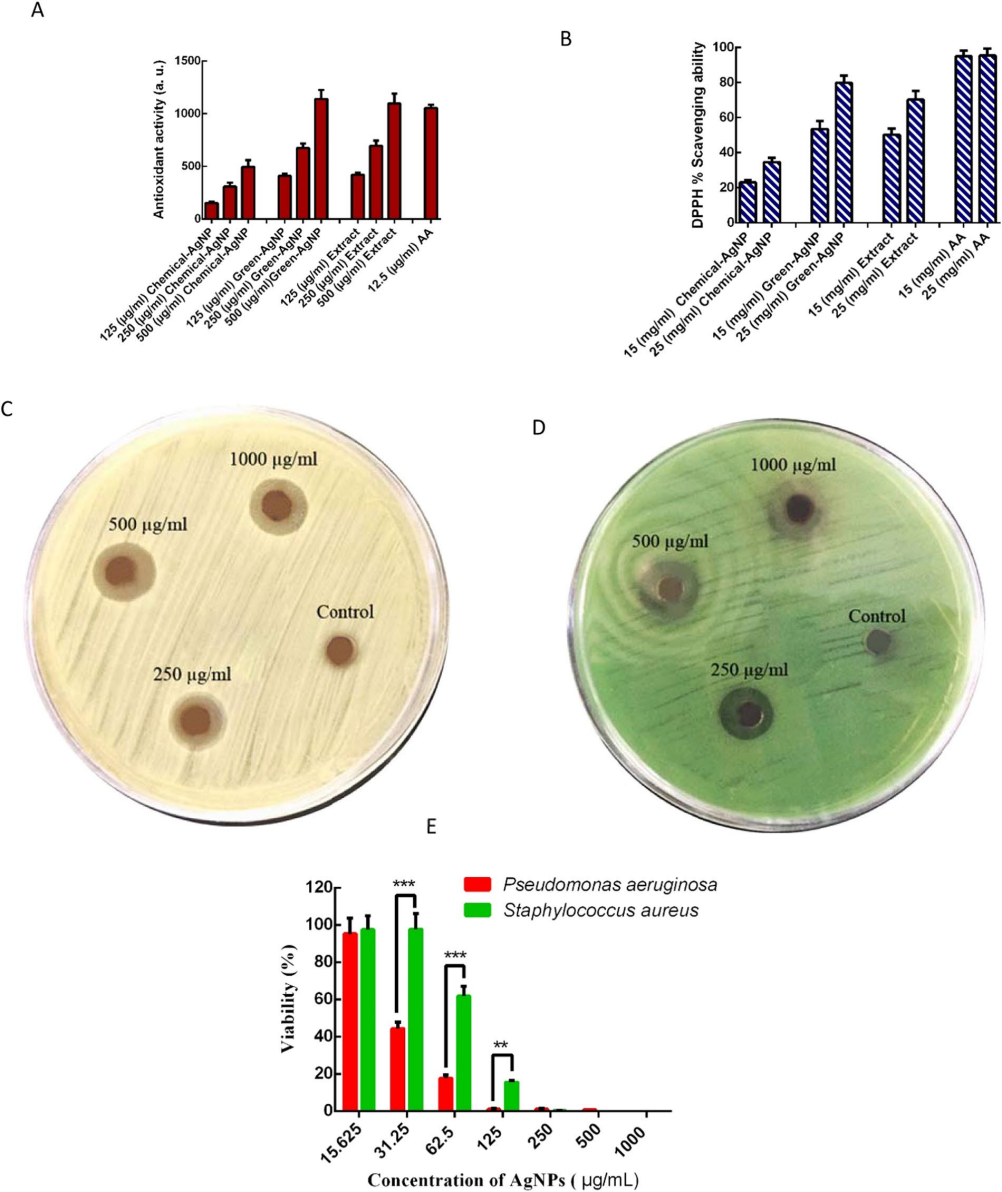
Antioxidant and Antibacterial activities of green synthesized AgNPs. The FRAP (**A**) and DPPH (**B**) radical scavenging capacity of AgNPs synthesized using *T. polium* extract. Data are expressed as mean ± SD of three independent (AA: ascorbic acid). (**C**) Antibacterial activity of green AgNPs against *S. aureus* was examined in the presence of paper discs that were soaked with different concentrations of AgNPs using the diffusion disc method. (**D**) Antibacterial activity of green AgNPs against *P. aeruginosa* was examined in the presence of paper discs that were soaked with different concentrations of AgNPs were examined using the diffusion disc method. (**E**) The survival of *S. aureus* and *P. aeruginosa* in the presence of different concentrations of AgNPs for 24 h in a 96-well plate. The date was reported as mean ± SD. (n =  3).

Cytolytic enzymes, free oxygen radicals and inflammatory mediators released by activated leukocytes at the wounded site, cause an imbalance between local pathological factors and integrity of immune defenses^51^. This encourages colonization of both Gram-positive and Gram-negative bacteria, where *S. aureus* and *P. aeruginosa* are the most predominant bacterial strains detected at wounded sites. Here, the antibacterial activity of biosynthesized AgNPs and chemically synthesized AgNPs was evaluated against these clinical antibiotic-resistant human pathogens, *S. aureus* and *P. aeruginosa*, using both disc diffusion and MTT assay^2^. *In vitro* antibacterial activity of biosynthesized AgNPs was first determined by agar disc diffusion assay. The clear zone of inhibitions (ZOI) was observed around discs loaded with AgNPs, meanwhile, no ZOI was observed around the control disc (Fig. 4c,d). These results clearly indicate that AgNPs showed a strong antimicrobial effect. The “bactericidal” activity of the as-formed AgNPs was further revealed using MTT assay; wells containing only AgNPs were taken as the control groups (Fig. S7). As shown in Fig. 4e, *S. aureus* and *P. aeruginosa* exhibited a concentration-dependent sensitivity toward biosynthesized AgNPs, where cell viability was reduced at higher concentrations of AgNPs. In other words, as shown in Fig. 4c–e the inhibitory effects of biologically synthesized AgNPs were observed at concentrations of 250 to 1000 µg/ml against *P. aeruginosa* and *S. aureus*, in a similar manner to the antibacterial activity of chemically synthesized AgNPs (Fig. S5). It should be noted that the maximum MIC values of AgNPs were observed at 250 µg/mL and 125 µg/mL for *S.aureus* and *P.aeruginosa*, respectively. Hence, the result of the antibacterial assay demonstrated that the green synthesized AgNPs similar to chemically synthesized AgNPs efficiently killed the gram-negative and gram-positive bacteria.

In the last decade, inorganic nanoparticles (NPs) have been extensively explored as antibiotic alternatives owing it to cost-effectiveness and powerful outcomes. Fortunately, the qualitative and quantitative analyses confirmed a concentration-dependent “bactericidal” activity of AgNPs. This type of nanoparticles has exhibited the positive effects on wound healing, suppressing local skin inflammation and preventing pathogens from entering the skin^52,53^. Mechanistically, silver is toxic to many bacterial components such as (i) cell wall where it causes transport blockage and plasma membrane collapse, (ii) enzymatic systems such as respiratory cytochromes, and (iii) microbial DNA and RNA where it prevents transcription and division^54,55^. It is unlikely that the bacterial cell will develop “AgNP” resistance since silver displays multiple antimicrobial mechanisms against the bacteria^56^. The present study clearly depicts the antibacterial activity of biosynthesized AgNPs against both Gram-positive and Gram-negative bacteria; a considerable inhibitory pattern was observed similar to previous reports^57,58^.

Furthermore, the synthesized AgNPs in presence of *T. polium* showed an appropriate antibacterial activity comparable to green synthesized AgNPs reported so far^59,60^. Additionally, AgNPs in this study showed a lower minimal inhibitory concentration toward *P.aeruginosa* compared to *S.aureus*. Rodrigues de Araujo *et al*. and Saravanakumar *et al*. also reported a higher antibacterial activity of green synthesized AgNPs against *P*. aeruginosa compared to *S. arouse*. The potential reasons for this observation may be attributed to the presence of dense layer of peptidoglycan on the surface of gram-positive bacteria, which undermines the penetration of AgNPs into bacteria and in turn, reduces their antibacterial activates^59,60^.

### The effect of biogenic AgNPs on macrophages cells

Despite their potent antibacterial activity and a wide range of biomedical applications, the use of AgNPs as therapeutic agents is limited because of their cytotoxicity against mammalian cells. On the other hand, there is considerable evidence regarding the critical role of macrophages in orchestrating the wound-healing process^61^. Hence, we assessed AgNPs’ cytotoxicity against macrophage-like cell line (RAW264) using MTT assay with various concentrations of AgNPs.

**Figure 5:**
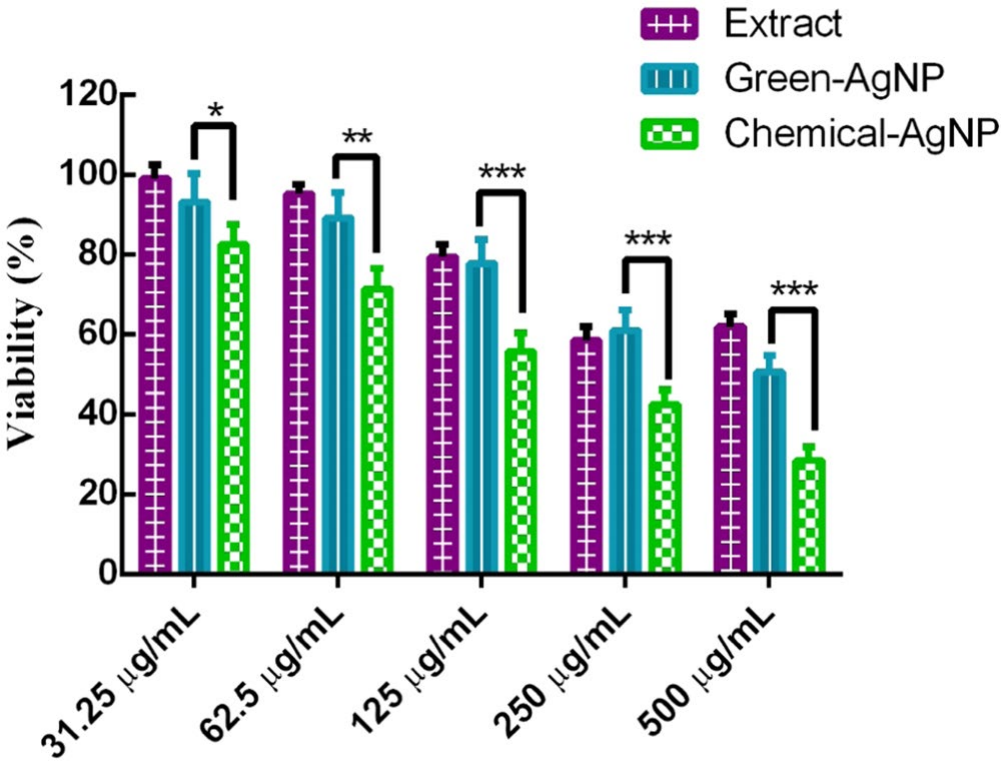
Cytotoxicity of AgNPs and extract of *T. polium* determined by using colorimetric MTT assay in macrophage-like cell line (RAW264) at different concentrations of samples. The date was reported as mean ± SD. (n =  3).

The percentage of viable cells decreased with increasing concentration of AgNPs (Fig. 5). Cell viability upon treatment with 500 µg/ml of *T. polium* extract, green-AgNP and chemically-AgNP was ~60%, ~50% and ~30%, respectively. However, upon treatment at concentrations of 31.25 and 62.5 µg/mL of green-AgNP, no significant cytotoxicity was observed, implying safe usage at these concentrations. These results demonstrated that green-AgNPs, in comparison with chemical-AgNPs, did not exhibit cytotoxicity against macrophage cells at concentration 62.5 µg/mL, while they are still retaining their bactericidal activity.

The green synthesized nanoparticles showed significantly higher selectivity index values compared to chemically synthesized AgNPs (Table S1). In other words, the results of antibacterial activity and biocompatibility assays of AgNPs demonstrated that the utilization of green synthesis method increased the selectivity of AgNPs for showing toxicity against *P.aeruginosa* and *S.aureus* relative to mammalian cells (Fig. S6A,B). This high selectivity index value of green synthesized AgNPs could be presumably explained by the presence of antioxidant materials on the surface of AgNPs. Moreover, using green synthesis method prevented using hazardous materials, which induce cytotoxicity in cells. Therefore, the green synthesis strategy increased the applicability of the AgNPs for killing the antibiotics-resistance bacterial pathogens.

### Mechanical, morphological and antibacterial properties of nanocomposite

**Figure 6:**
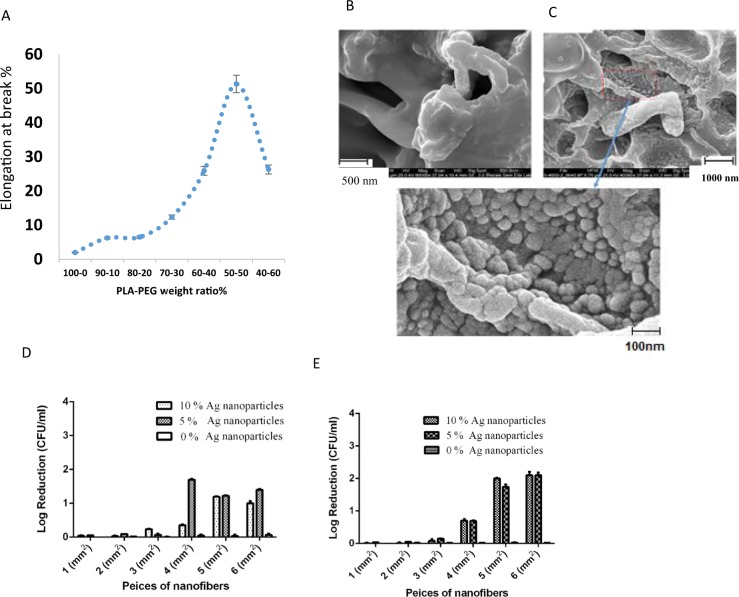
Fabrication of Ag nanofilm and evaluation of its antibacterial activity. (**A**) Tensile test for prepared PLA/PEG at various ratios (**B**) SEM micrographs of PLA/PEG nanofibers without AgNPs. (**C**) SEM micrographs of Ag nanofilm. (**D**) Antibacterial activity Ag nanofilm containing AgNPs against *S. aureus*. (**E**) Antibacterial activity of Ag nanofilm against *P. aeruginosa*. The date was reported as mean ± SD. (n =  3).

Polylactic acid (PLA) is one of the most widely used biodegradable thermoplastic polyesters in clinical applications. This bioplastic largely due to the favorable biocompatibility of the polymer and its safe degradation products, has captured a great attention. Upon contacting biological media, the polymer starts to disintegrate into lactic acid (LA) or to carbon dioxide and water; products that are metabolized normally by the cells and don’t impose any harm. The degradation of PLA is further enhanced by enzymes secreted by a bacterial infection or inflammation cells. Disintegration takes place both on the surface and inside the polymer through water diffusion between polymer chains^62^. However, features such as poor toughness, low degradation rate, and high hydrophobicity limit the implementation of PLA in wound dressings. One of the approaches to facilitate the degradation of PLA is increasing porosity of the polymer, where it would be easier for water or enzyme molecules to diffuse through polymer chains^63^. In this study, blending aimed at obtaining a composition with desirable properties to produce absorbable wound dressing, therefore, the final blend should have a proper strain for better control and handling of the wound. According to the results of the tensile test and taking into account the above criterion, PLA/PEG sample with 50 wt% PEG had the better mechanical properties and selected for further biological examinations (Fig. 6a).

PLA blending with polyethylene glycol (PEG) results in a porous film; porosity is very crucial for a wound dressing because it affects other properties of dressing including water vapor transmission rate, oxygen transmission rate. Furthermore, dressings with smaller pore size are preferable in order to avoid trauma/pain on dressing change^64^. The morphology of AgNPs-nanofilm was examined using scanning electron microscope (SEM); clear porous, sponge-like network of PLA/PEG nanofibers can be observed (Fig. 5b). The formation of pores can be attributed to interfacial tension instability, dependent on the organization of the polymer at the interface during the emulsion evaporation process^65^. The distribution of PLA/PEG nano/microfibers by the diameter is depicted in Fig. 6b. The SEM analysis showed uniform dispersion of AgNPs in the PLA/PEG nanofiber composite, with a size in the range of 61.4–90.26 nm (Fig. 6c). Furthermore, as shown in Fig. S1 the mean pore size of the nanofilm was estimated to be about 10 µm. Interestingly, the EDS mapping analysis confirmed the presence and the uniform distribution of AgNPs within the nanofilm with no evident agglomeration (Supplementary Fig. S2A,B). Additionally, according to the elemental analysis of EDX-SEM, AgNPs percentage in the film was estimated to be ~3.14% (Supplementary Fig. S2).

Figure S3A,B show the FTIR spectra of the PLA/PEG nanofilms without and with entrapped AgNPs, respectively. It can be found that the both spectrums have almost the same absorption peaks which are perfectly matched with the previous PLA/PEG FTIR reports^45,66,67^. At the same time, several slight shifts for the entrapped AgNPs sample are related to the interaction of Ag NPs with the PLA/PEG composite. In detail, the shift of the C–O stretching frequency from 1084.5 cm-1 (for PEG–PLA sample) to 1087 cm-1 (for PEG–PLA@Ag sample), and the strong decrement in the intensity of the –CH2 stretching frequency of the PEG–PLA@Ag sample (in comparison with that of PEG–PLA sample) with its slight shift from 2875.2 cm-1 to 2877.6 cm-1, are attributed to the conjugation of oxygen atoms with the surface of AgNPs^45,67^.

Thermogravimetric analysis (TGA) curves provided the information about material degradation and thermal stability of free and Ag loaded nanofilms. In both cases the existence of PEG as a plasticizer has decreased the thermal stability of nanofilm. However, loss of weight in the unloaded nanofilms was higher in comparison with the nanocomposite. However, two T50% °C at about 250 °C and 350 °C for unloaded fiber converged to T50% °C at 300 °C for nanocomposite, influenced by AgNPs presence. Hence, the presence of AgNPs boosted the resistance to degradation from 350 °C to 500 °C in AgNPs nanofilm.

Furthermore, the potential of AgNPs nanofilm as functional wound dressings was assessed by evaluating their antibacterial activity against bacterial strains found on wounds. For this purpose, the antibacterial activity of free and AgNPs nanofilm was evaluated on *P. aeruginosa* and *S. aureus* cultures. As shown in Fig. 6d,e, growth rates dropped in *P. aeruginosa* and *S. aureus* after being exposed and incubated with PLA/PEG nanofibers loaded with various concentrations of biogenic AgNPs. PLA/PEG nanofibers 5 and 6 mm^2^ in dimension, loaded with 5% and 10% AgNPs, exhibited strong antibacterial activity against both bacterial strains. The antibacterial activity Ag nanofilm declined along with the decrease of the dimensions of the Ag nanofilm.

On the other hand, sheet of PLA/PEG nanofibers 4 mm^2^ in dimension, loaded with 5% and 10% AgNPs, exhibited similar antibacterial activity against *P.aeruginosa*. However, a different antibacterial pattern was observed against *S. aureus*, where PLA/PEG nanofibers loaded with 5% AgNPs exhibited complete inhibition of bacterial growth whereas those loaded with 10% AgNPs exhibited antibacterial activity to a lesser extent. The antibacterial activity is attributed to the release of AgNPs loaded onto PLA/PEG nanofibers into the media; since plain PLA/PEG nanofibers possess no inhibition potential against the examined bacteria. In the next step, we have evaluated the release of AgNPs from PLA/PEG/AgNPs nanofilm. As shown in Fig. S8, Uv-vis analysis demonstrated continuous increase of SPR absorbance of samples as a function of time, indicating the sustained release of AgNPs from PLA/PEG/AgNPs nanofilm. This result was consistent with suitable antibacterial activity of PLA/PEG/AgNPs compared to AgNPs unloaded PLA/PEG.

A number of studies have incorporated the antibacterial compound into PLA/PEG polymer for various applications. It has been reported that, incorporation of magnesium oxide nanoparticles into PLA/PEG film enhanced antibacterial efficacy of the film. However, in comparison to our study, less than 50% bactericidal activity has been reported after 24 h of treatment^34^. Moreover, chemically synthesized Ag nanoparticles deposition was also reported by Turalija *et al*. to enhance antibacterial property of PEG/PLA films^35^, but a slightly lower antibacterial efficacy against *S.aureus* has been observed compared to our study. Moreover, the cytotoxicity of chemically synthesized Ag nanoparticles was the Achilles heel of such a design, which was confirmed in our study.

Other studies have also integrated natural antioxidant agents like α-tocopherol, Umbelliferone and rosemary extract to the PLA film directly. For example, it has been demonstrated that the incorporation of α-tocopherol as a natural antioxidant into PLA film led to about 6-fold enhancment of radical scavenging activity of the film^31^. Moreover, antioxidant property of PLA bilayer films containing Umbelliferone has been increased by incorporation of lignin nanoparticles^32^. Vasile *et al*. prepared PLA/PEG containing rosemary extract as antioxidant agent and chitosan as antimicrobial agent for subcutaneous implantation^36^. In spite of suitable antioxidant activity of rosemary extract, the antibacterial activity of chitosan against *S.aureus* was about 100-fold, lower than that of our biogenic AgNPs.

In comparison to mentioned studies, the unique advantageous of our design is using biogenic AgNPs to simultaneously endow antibacterial and antioxidant properties to PLA/PEG film, without cytotoxicity challenges. Whereas, as mentioned above other reported designs required incorporation of distinct antioxidants and antimicrobials compounds separately to improve PLA/PEG film properties for biomedical application. Generally, according to our results, the challenges associated to wound healing process are expected to overcome using this novel biogenic nanofilm with efficient antioxidant and antimicrobial properties.

## Conclusions

The novel strategy of using PLA/PEG nanofilm carrying a reservoir of biogenic AgNPs in the form of composite nanofiber membranes as a wound dressing has shown promising outcomes. Initially, using *T. polium* extract as a reducing agent, biocompatible AgNPs were synthesized and characterized well by various techniques. This green synthesis approach proved to be rapid, cost-effective and an efficient way for the synthesis of AgNPs, which exclude external stabilizers/reducing agents. Biogenically synthesized AgNPs exhibited proper antibacterial and antioxidant activity. The cytotoxic assessment further confirmed the safety of utilizing the biogenically synthesized AgNPs in macrophages, since it was demonstrated that a concentration of AgNPs (62.5 μg/mL) could be implemented exhibiting antibacterial activity with no significant cytotoxic effects. Subsequently, Ag/PLA/PEG nanofilms with 5 and 10 wt% were prepared and exhibited significant antimicrobial activity against both against *P. aeruginosa* and *S. aureus*. Taken together, this novel Ag nanofilm with simultaneous antimicrobial and antioxidant properties has strong potential as a wound dressing, however, “*in vivo*” assessments should be conducted to further confirm the safety and functionality of the designed wound dressing film.

## Supplementary information

Supplementary Information.
