# Supplementary material for: Facile preparation of a novel biogenic silver-loaded Nanofilm with intrinsic anti-bacterial and oxidant scavenging activities for wound healing
Source: Sci Rep. 2020 Apr 9;10:6129. doi: 10.1038/s41598-020-63032-5 (PMC7145826; doi:10.1038/s41598-020-63032-5)
Supplement: Supplementary file 1 — Supplementary Information. [file 41598_2020_63032_MOESM1_ESM.docx]

Supportive Information

Facile preparation of a novel biogenic silver-loaded Nanofilm with intrinsic anti-bacterial and oxidant scavenging activities for wound healing

Hassan Bardania^1,2,3^, Reza Mahmoudi^1^, Hamed Bagheri^4^, Zeinab Salehpour^2^, [Mohamad Hassan Fouani](http://www.ijbiotech.com/?_action=article&au=330401&_au=Mohamad+Hassan++Fouani)^5^ , Bita Darabian^4^ , Seyed Sajad Khoramrouz^1^, Ali Mousavizadeh^6^, Majid Kowsari^7^, Seyyed Ebrahim Moosavifard^7^, Gunna Christiansen^8^, Danesh Javeshghani^7^, Mohsen Alipour^7^*, Mohammad Akrami^9^*

1-Cellular and Molecular Research Center, Yasuj University of Medical Sciences, Yasuj, Iran.

2-Medicinal Plant Research Center, Yasuj University of Medical Sciences, Yasuj, Iran.

3-Clinical Research Development Unit, Imamsajad Hospital, Yasuj University of Medical Sciences, Yasuj, Iran.

4- Faculty of Interdisciplinary Science and Technology, Tarbiat Modares University, Tehran,Iran

5-Department of Nanobiotechnology, Faculty of Biological Sciences, Tarbiat Modares University, Tehran, Iran

6- Social Determinants of Health Research Center, Yasuj University of Medical Sciences, Yasuj, Iran.

7-Department of Advanced Medical Sciences & Technologies, School of Medicine, Jahrom University of Medical Sciences, Jahrom, Iran

8-Department of Biomedicine, Aarhus University, 8000 Aarhus C, Denmark.

9-Department of Pharmaceutical Biomaterials and Medical Biomaterials Research Center, Faculty of Pharmacy, Tehran University of Medical Sciences, Tehran, Iran.

*Corresponding Authors: Mohsen Alipour [m.alipour@jums.ac.ir](mailto:m.alipour@jums.ac.ir) Tel: +98 7154331520 and Mohammad Akrami [m-akrami@sina.tums.ac.ir](mailto:m-akrami@sina.tums.ac.ir)


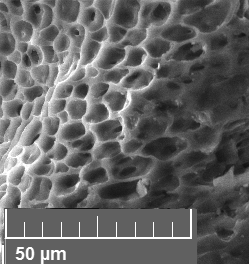


**Fig. S1**: SEM image of PLA/PEG/AgNPs Nanofilm

**Elemental analysis and EDS mapping of Ag nanofilm**

To investigate the elementary compositions and silver distribution of the film, Field emission scanning electron microscopy equipped with Energy-dispersive spectra (FESEM-EDS); (TESCAN, MIRA III version, France) and FESEM with energy dispersive X-ray spectroscopy (EDX) was performed. The image of sample section was obtained at 15keV and acquisition period of 120 s.

**Release of green synthesized silver nanoparticles from PLA/PEG/Ag nanofilm**

Release of green synthesized silver nanoparticles from PLA/PEG/Ag nanofilm was evaluated by Uv- vis analysis. A piece of Ag-nanofilm (1 × 0.5 cm) with 10 % AgNPs was placed in 1.5 ml of phosphate buffer saline and incubated in a shaker incubator with 150 rpm and 37 °c for 96 h. the release Ag nanoparticles were evaluated by periodic sampling and analysis of absorbance of nanoparticles by Uv-vis analysis.

## Selectivity index of nanoparticles

The capability of green Ag nanoparticles for inhibition of P. aeruginosa growth and their cytotoxicity against the human cell line were examined at different concentrations. Selectivity index of nanoparticles (green synthesized and chemically synthesized) was calculated with following formula: 50% toxicity in cell/ 50% toxicity in bacteria *100%.


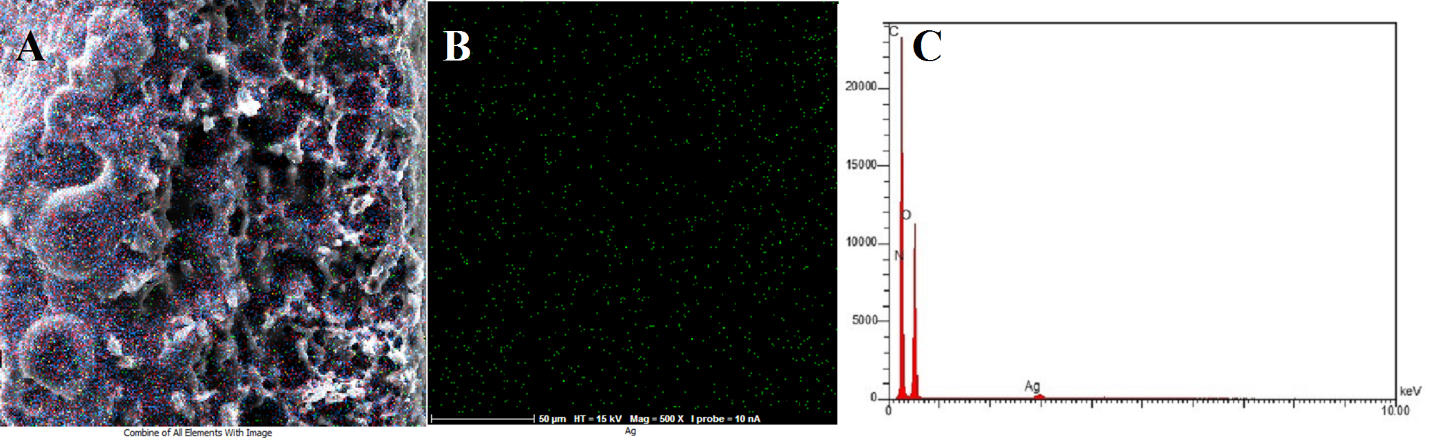


Fig. S2. EDS mapping (A and B) and EDX analysis © of Ag nanofilm


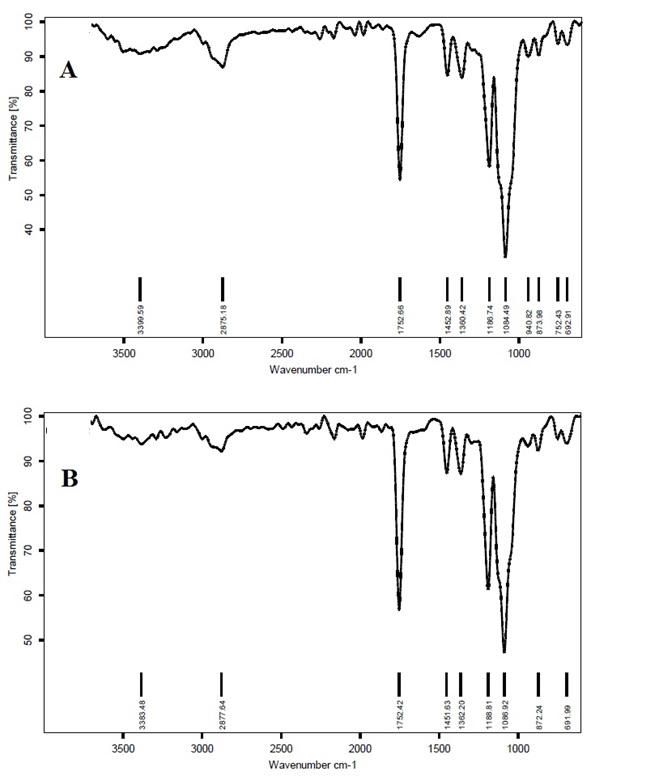


OH stretching

Figure S3: FTIR spectra of (A) AgNP/extract loaded and (B) unloaded nanofilm


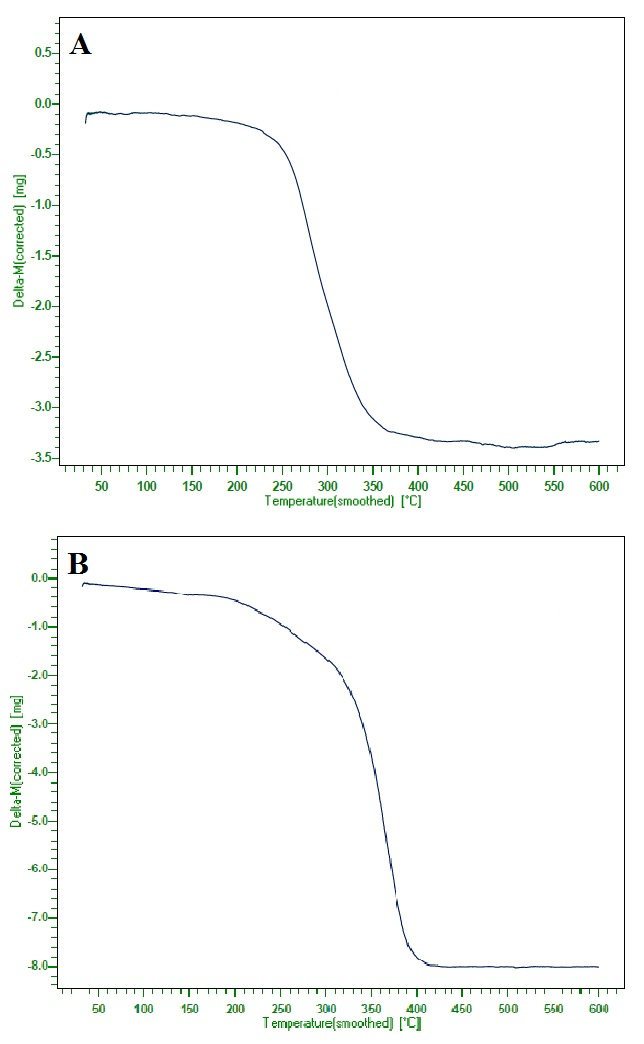


Figure S4: Thermogravimetric analysis (TGA) of AgNP/extract loaded (A) and unloaded (B) nanocofilm


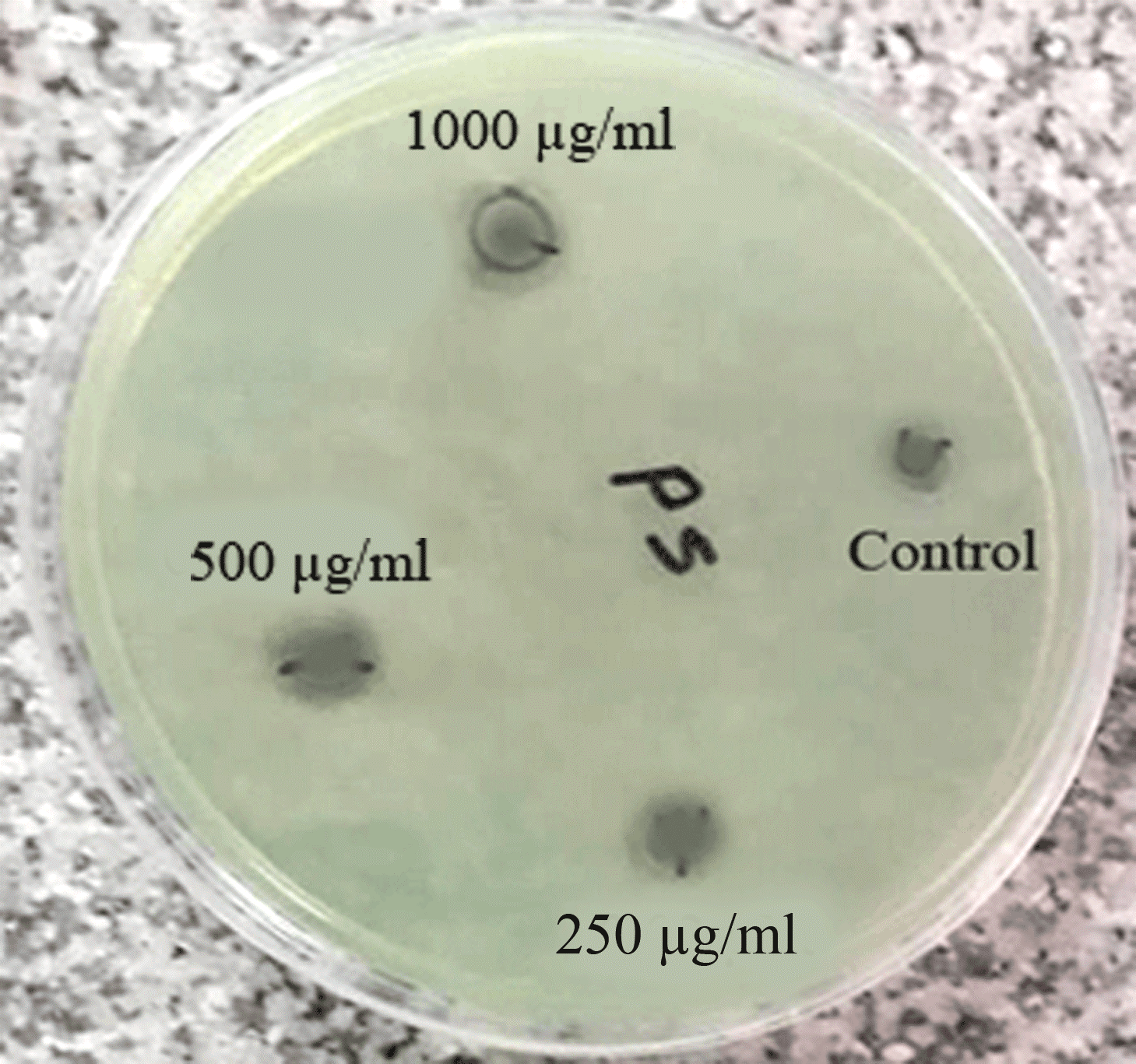


A B


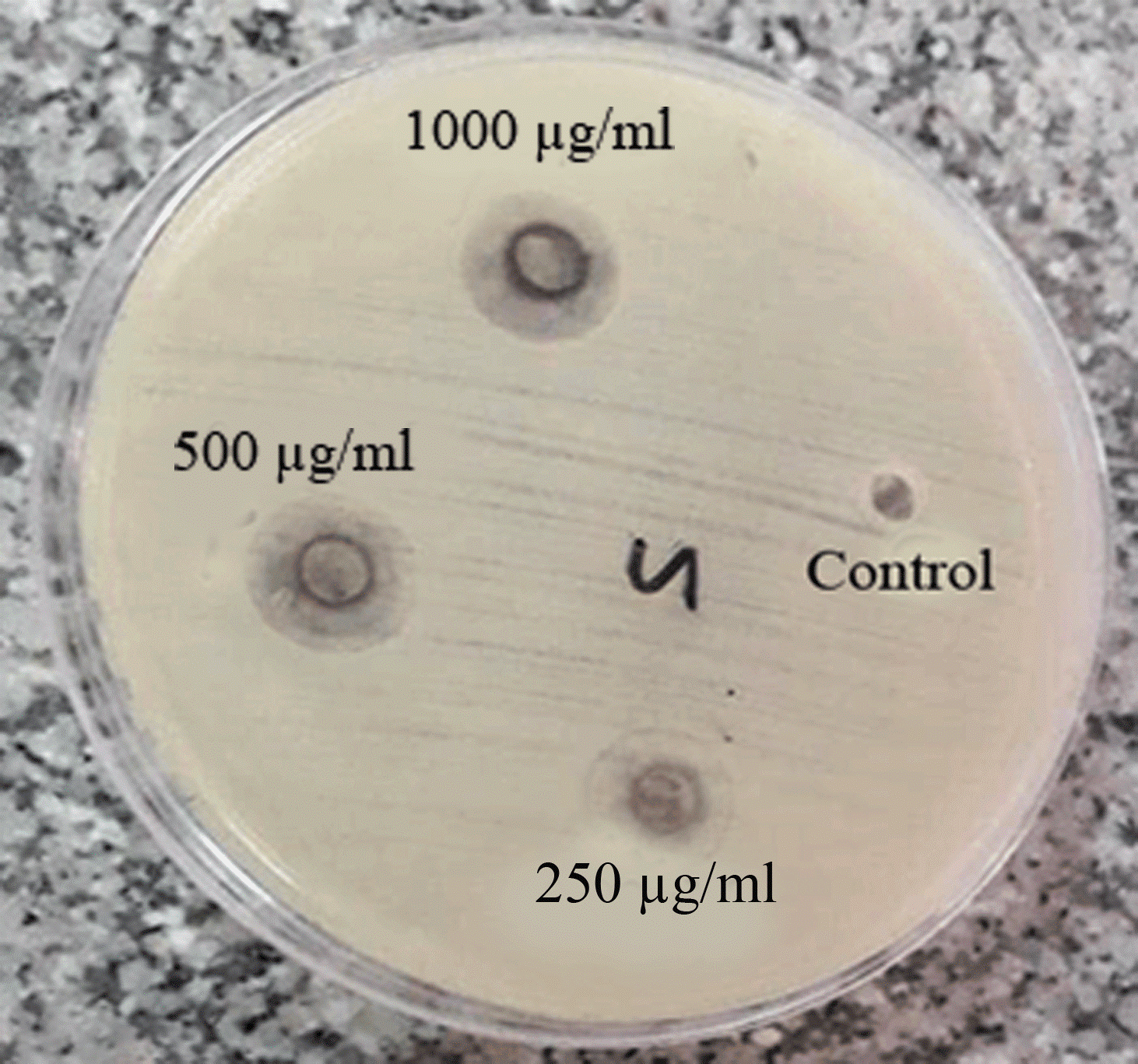


C


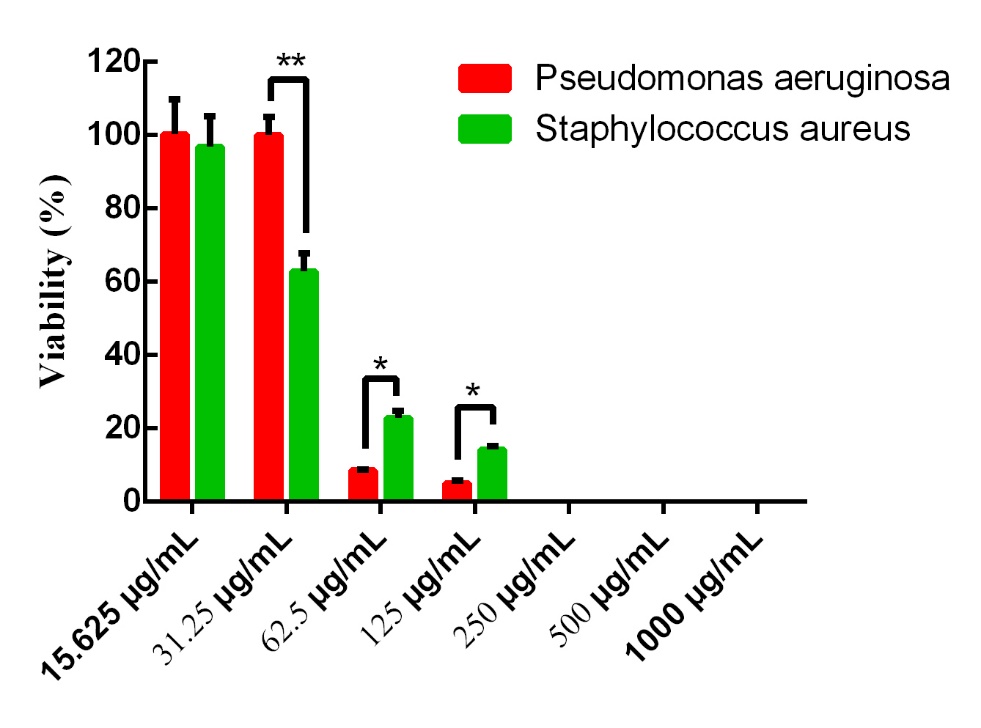


Figure S5 Antibacterial activity of chemically synthesized AgNPs A) Antibacterial activity of chemically synthesized AgNPs against *P. aeruginosa* growth was examined in presence of paper discs that were soaked with different concentrations of AgNPs using the diffusion disc method. B) Antibacterial activity of chemically synthesized AgNPs against *S. aureus* was examined in presence of paper discs that were soaked with different concentrations of AgNPs using the diffusion disc method. C) Quantitative analysis of the effect of chemically synthesized AgNPs on with various concentrations of on the survival of P. aeruginosa and S. aureus for 24 h in a 96-well plate. The date was reported as mean ± SD. (n = 3)

A

B)

Figure S6: Antibacterial activity and toxicity profiles of nanoparticles A) Antibacterial activity and toxicity of green synthesized Ag nanoparticles. B) Antibacterial activity and toxicity of chemically synthesized Ag nanoparticles.

Figure S7. MTT assay of AgNPs without bacteria at different concentrations. The date was reported as mean ± SD. (n = 3)


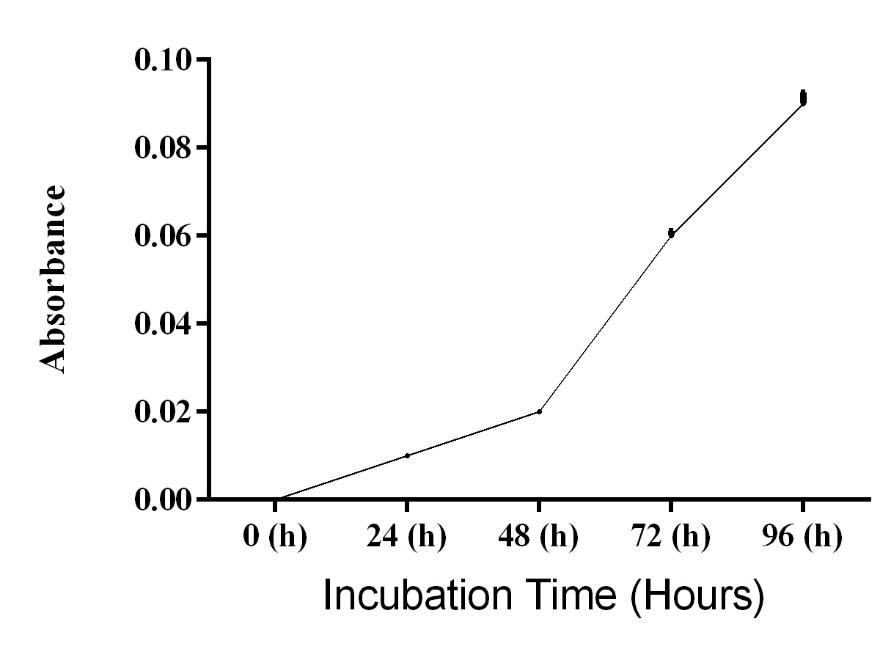


Figure S8. Cumulative Release of Ag nanoparticle from PLA/PEG/Ag nanofilm as a function of time. The release of Ag nanoparticles into PBS solution during four days was measured by Uv- vis analysis.


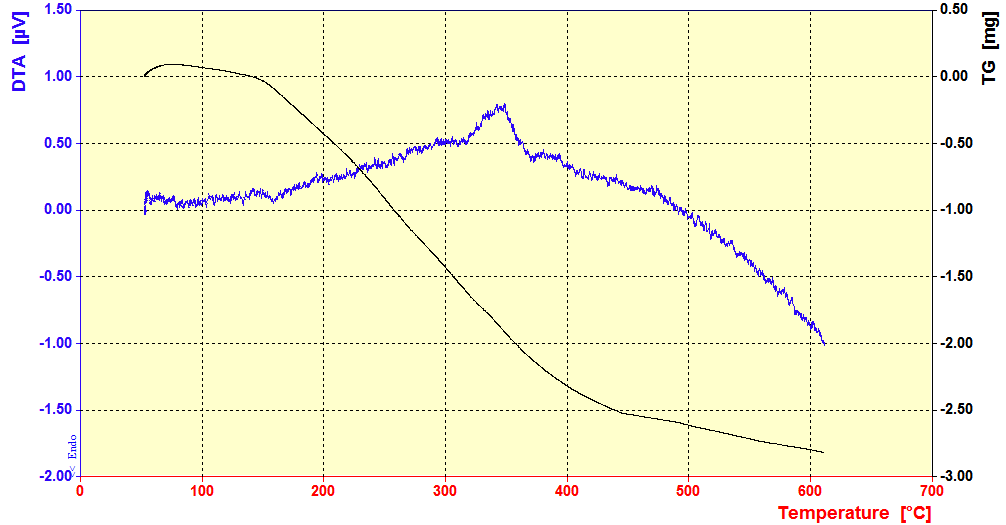


Figure S9. Simultaneous Thermogravimetric and differential thermal analysis (TG/DTA) of green synthesized AgNPs.

Table S1 Table Selectivity index values of AgNPs against bacterial pathogens

|  |  | 50% Toxicity *(*µg/mL*)^a^* |  | selectivity index ^b^ | | |  |
| --- | --- | --- | --- | --- | --- | --- | --- |
|  | *P.aeruginosa* | *S.aureus* | RAW264 cell | *P.aeruginosa* | | *S.aureus* | |
| Green AgNPs | 30 | 74 | 500 | 1666 | 675 | |  |
| **Chemical AgNPs** | 48 | 45 | 175 | 364 | 388 | |  |

a Concentration resulting in 50% inhibition of growth of bacterial and mammalian cells

b Selectivity index value calculated as (50% Toxicity in RAW264 cell /50% Toxicity against .)*100 %
